# Exploring the Potential of *Aspergillus oryzae* for Sustainable Mycoprotein Production Using Okara and Soy Whey as Cost-Effective Substrates

**DOI:** 10.3390/jof10080555

**Published:** 2024-08-07

**Authors:** Putu Virgina Partha Devanthi, Ferren Pratama, Ihsan Tria Pramanda, Mario Donald Bani, Adinda Darwati Kadar, Katherine Kho

**Affiliations:** Indonesia International Institute for Life Sciences, Pulomas Barat Kavling 88, Jakarta 13210, Indonesia; ferren.pratama@alumni.i3l.ac.id (F.P.); ihsan.pramanda@i3l.ac.id (I.T.P.); mario.bani@i3l.ac.id (M.D.B.); adinda.kadar@i3l.ac.id (A.D.K.); katherine.k@i3l.ac.id (K.K.)

**Keywords:** mycoprotein, *Aspergillus oryzae*, filamentous fungi, tofu by-products, soy by-product, submerged fermentation, alternative protein, circular economy

## Abstract

Mycoprotein is an alternative protein produced through fungal fermentation. However, it typically relies on refined glucose syrup derived from starch, which can be costly and unsustainable. This study investigates the potential of soybean processing by-products (okara and soy whey) as alternative substrates for producing mycoprotein using *Aspergillus oryzae*. *A. oryzae* was cultured for 7 days at 30 °C in diluted okara (1:50) and soy whey (1:1) with or without agitation (100 rpm). Soy whey produced higher biomass yields (369.2–408.8 mg dry biomass/g dry substrate), but had a lower biomass concentration (0.783–0.867 g dry weight/L). Conversely, okara produced a higher biomass concentration (2.02 g dry weight/L) with a yield of 114.7 mg dry biomass/g dry substrate. However, biomass formation in okara was only observed in static conditions, as agitation caused biomass to entangle with soy pulp, hampering its production. Additionally, okara tended to release protein into the media, while soy whey accumulated protein within the biomass, reaching up to 53% *w*/*w* protein content. The results of this study provide a promising approach to addressing both soybean processing waste reduction and food security concerns.

## 1. Introduction

Protein is an indispensable part of a healthy diet, playing a key role in maintaining and repairing the body’s tissues while supporting various metabolic functions. However, the reality is that many low-income communities struggle to access affordable and nutritious protein sources, with animal-based proteins such as meat and dairy generally being expensive [1,2]. This limited access has severe consequences, leading to widespread malnutrition, increased susceptibility to diseases [3], and stunted growth, particularly in children [4]. The situation worsens when considering the production of animal-based protein, which heavily relies on resource-intensive livestock farming and is environmentally unsustainable; it leads to deforestation [5], habitat destruction [6], and increased greenhouse gas emissions [7]. As the global population continues to grow, the demand for protein is expected to rise [8], further straining these unsustainable practices and necessitating a shift towards more sustainable solutions.

In recent years, plant-based protein has been increasingly promoted and perceived as a superior alternative to traditional meat due to its lower ecological footprint. However, its production still imposes a burden on arable land and potable water resources [9]. Additionally, they often contain fewer essential amino acids than animal-based proteins as well as anti-nutritional factors that may impede protein digestion and absorption [10,11]. In contrast, recycling animal-based waste such as crustacean shells through biorefinery processes could provide a more sustainable high-quality source of protein [12]. Nonetheless, the availability of crustacean shells can vary depending on season and region, making it necessary to explore alternatives for a consistent supply throughout the year and across regions.

Microbial-based proteins offer several advantages over other alternative protein sources, such as minimal land and water requirements, independence from seasonal fluctuations, and high nutritional quality [10]. Additionally, microbes can thrive on a wide range of inexpensive substrates, such as food waste and agricultural residues, potentially making their production more environmentally friendly and cost-effective [13,14]. Microalgae (e.g., *Spirulina*) are popular microbial sources with up to 80% protein content and amino acid profiles that match dietary requirements [15]. However, their unappealing taste may discourage consumption, potentially leading to reduced protein intake [16]. On the other hand, mycoprotein derived from fungi stands out due to its meat-like structure and sensory characteristics closely resembling conventional meat [17,18]. It has a protein content of 50–55% and contains all the essential amino acids needed for muscle growth and development. Mycoprotein’s bioavailability is comparable to milk, with a Protein Digestibility Corrected Amino Acid Score (PDCAA) of 0.996 [19,20,21]. Additionally, a systematic review by Derbyshire and Delange [22] shows a correlation between acute consumption of mycoprotein and reduced cholesterol levels.

Mycoprotein is produced through a fermentation process, where fungal mycelium is cultivated in controlled environments using carbohydrates, mainly glucose, and oxygen [23]. Nutrients are converted into protein-rich biomass, which can be harvested, purified, and processed into various food products [19]. The most well-known mycoprotein brand available in the market is currently sold under the Quorn™ brand (https://www.quorn.co.uk/, accessed on 10 November 2023). The company produces mycoprotein by cultivating *Fusarium venenatum* in large bioreactors, however, this process can be quite expensive as it requires highly refined glucose syrup as a carbon source [23]. Additionally, the production media for *F. venenatum* mycoprotein is supplemented with ammonium and biotin [23,24,25], further increasing its cost and resulting in a market price similar to meat.

While mycoprotein production has long centered around *F. venenatum*, the landscape has recently evolved, as researchers are increasingly exploring the potential of alternative fungal species. This shift has been motivated mainly by the opportunity to convert various agricultural and industrial byproducts that would otherwise be discarded or considered waste, into valuable protein-rich biomass through mycoprotein production [26,27,28,29]. Among the studied filamentous fungi, *A. oryzae* has shown great promise due to its versatility and high protein yield. For instance, a study conducted by Souza-Filho et al. [26] demonstrated that *A. oryzae* achieved the highest mycoprotein yield from pea processing by-products, at 0.26 g/g, surpassing *F. venenatum*, *Monascus purpureus*, *N. intermedia*, and *Rhizopus oryzae*. Karimi et al. [27] found that *A. oryzae* achieved the highest biomass yield, reaching 103.0 ± 2.7 g dry mass/L, 1.3 and 3.7 times greater than *N. intermedia* and *R. oryzae*, respectively. Furthermore, Gamarra-Castillo et al. [30] successfully produced *A. oryzae* biomass with a protein concentration of up to 17% using maltodextrin as a carbon source, higher than the protein content of Quorn™ (12.8–14.5%). An even higher protein content in the biomass, reaching up to 37% dry mass, was achieved by cultivating *A. oryzae* in oat flour through submerged fermentation in an airlift bioreactor [31].

For centuries, *A. oryzae* has also played a crucial role in the traditional fermentation of soybean-based foods, such as soy sauce and soybean paste (*miso*) [32]. Its longstanding use is attributed to its remarkable enzymatic capability, allowing it to effectively break down complex molecules like carbohydrates and proteins found in soybeans [33,34]. This knowledge has motivated us to explore the feasibility of cultivating *A. oryzae* using soybean waste streams, including okara and soy whey, for mycoprotein production. Okara or soy pulp is a byproduct of soymilk and tofu production. It is widely available in Asian countries with high soybean consumption, and in 2019 alone, global production of okara reached around 14 million tons [35,36]. Okara is rich in nutritional content, containing around 40–60% dietary fiber (cellulose and hemicellulose), 4–5% fermentable carbohydrates (e.g., glucose, fructose, sucrose, raffinose, and stachyose), 15–33% protein, and 8–11% lipids per 100 g of dry weight [35]. However, it is usually used as animal feed or discarded due to its perishable nature [37]. To enhance the value of okara, researchers have explored the use of microbial transformation, including fungal fermentation with *Aspergillus*. For instance, *Aspergillus* sp. HK-388 was employed to ferment okara, producing 8-hydroxy daidzein, while *A. oryzae* was used to convert okara into okara-*koji* flour, okara-*miso*, and okara-*meju*, as summarized in a review by Vong and Liu [35]. Meanwhile, soy whey is a byproduct of tofu and soy protein isolate (SPI) production [38]. Similar to okara, soy whey still retains some nutrients from soymilk, including approximately 1% of carbohydrates (mainly stachyose and sucrose), 0.1–0.8% of proteins, 0.4–1.0% of fats, and 0.4% of minerals [38]. While okara is commonly utilized as animal feed, soy whey is often discarded, which can create a significant problem given its high organic composition. Disposing of it untreated not only results in the loss of valuable nutrients but also poses environmental risks by increasing chemical oxygen demand (COD) and biological oxygen demand (BOD) levels. Various biotransformation methods have been explored to enhance the value of soy whey, with a predominant focus on lactic acid bacteria (LAB). These methods include using soy whey as a growth medium for the propagation of LAB or using LAB to ferment soy whey into functional beverages [38].

Despite the potential advantages, there remains a significant gap in the literature concerning mycoprotein production using *A. oryzae* and soybean waste as substrates. To address this gap, our current investigation is centered around the research question of whether *A. oryzae* (isolated from *koji* starter culture) can efficiently grow in okara and soy whey to yield protein-rich biomass.

## 2. Materials and Methods

### 2.1. Substrate and Reagents

Potato dextrose agar (PDA) (Merck, Darmstadt, Germany) was used to grow and maintain the *A. oryzae* isolate used in this study. Skim milk (SM) agar was prepared according to the manufacturer’s instructions (Himedia, Mumbai, India). Starch agar was prepared consisting of 10 g/L starch, 3 g/L yeast extract, 5 g/L bacteriological peptone, and 15 g/L agar as described by Cappuccino et al. [39]. Tween 80 agar was prepared according to Yassein et al. [40] and contained 10 g/L bacteriological peptone, 5 g/L sodium chloride, 0.1 g/L calcium chloride dihydrate, and 15 g/L agar. Nutrient broth and agar (Merck, Darmstadt, Germany) were used to maintain *Pseudomonas aeruginosa,* which was used as a positive control in screening amylase, protease, and lipase. Soy pulp or okara was kindly provided by a local soy milk merchant based in Jakarta, Indonesia, and soy whey was also provided by a small-scale tofu producer located in Bekasi, Indonesia.

### 2.2. Microorganism Maintenance and Media Preparation

*A. oryzae* isolate (code: KKC.P0N.A) was obtained from the Indonesia International Institute for Life Sciences (i3L)’s culture collection. The isolate was previously obtained from commercially available koji starter culture (GEM cultures, Lakewood, WA, USA). Stock cultures of *A. oryzae* were maintained on PDA and stored at 4 °C. The inoculum used for mycoprotein production was freshly prepared by transferring a fragment of mycelium onto fresh PDA and then allowing it to incubate for 7 days at 30 °C. The fungal spore suspension was obtained by flooding the PDA cultures with 15 mL of 0.85% NaCl solution, followed by gently scraping off the spores using a sterile 100 μL micropipette tip. The spore concentration was then adjusted to ~log 6 spores/mL and immediately used. *P. aeruginosa* was also obtained from i3L’s culture collection and cultured in NB from stock culture at 30 °C for 24 h before enzymatic analysis. 

Prior to media preparation, okara was dried at 60 °C in an oven until the weight was constant. Then, okara media was prepared by dissolving 0.88 g of dried okara into 44.12 mL of distilled water (1:50 ratio). Separately, 45 mL of soy whey media was prepared by mixing soy whey with distilled water at a 1:1 ratio. Both media were autoclaved at 121 °C for 15 min and stored at 4 °C until use. 

### 2.3. Enzymatic Activity of Aspergillus oryzae

To detect the proteolytic, amylolytic, and lipolytic activities in *A. oryzae* rapidly, standard plate assays with skim milk agar, starch agar, and Tween 80 agar, respectively, were used [39]. *Pseudomonas aeruginosa* served as the positive control [39,40]. First, *A. oryzae* spore suspension was prepared as mentioned in Section 2.2. for the assays. Then, 10 μL of the spore suspension was transferred into each well made in the agar plates [41,42]. The plates were incubated for 24 h at 30 °C, and the zone of activity was then measured using a vernier caliper and recorded in mm. For amylase activity, Gram’s iodine was added to the starch agar plates prior to measurement. 

Furthermore, the cellulolytic activity of *A. oryzae* was investigated using a K-CELL4V endoglucanase kit (Megazyme, Bray, Ireland) according to the manufacturer’s instructions. The spore suspension prepared according to Section 2.2. was grown in okara media for 7 days at 30 °C under static conditions and the filtrate was then subjected to cellulase activity testing. Cellulase from *Trichoderma* sp. provided in the kit served as a positive control.

### 2.4. Mycelial Biomass Production in Media Containing Soy By-Product

Two types of soy by-products were utilized to cultivate *A. oryzae* biomass: okara and soy whey. *A. oryzae* cultivation was done in 250 mL Erlenmeyer flasks, each containing 45 mL of the soy by-products and 5 mL of the spore suspension (~log 6 spores/mL). The cultures were then incubated for 7 days at 30 °C with agitation at 100 rpm. To better understand the impact of agitation on biomass and protein, another set of cultures were incubated statically under the same temperature conditions. The cultivation was carried out in triplicates for each media and condition.

At the end of cultivation, the cultures were filtered using Whatman paper to separate the retentate, comprising either fungal biomass only (for soy whey) or fungal biomass with soy pulp (for okara). The retentate was rinsed thrice with distilled water before further analysis. The liquid that passed through the filter was referred to as filtrate. In okara cultures, biomass was separated from soy pulp only when they were not entangled. When separable, the biomass floated on the surface while the soy pulp remained at the bottom. The biomass was then simply removed and rinsed to separate it from the soy pulp. When the biomass was entangled with the soy pulp, they could not be separately determined.

### 2.5. Analytical Methods

Samples were taken before and after the cultivation period for analysis. The retentate was evaluated based on its protein content and dry weight, while the filtrate was analyzed for pH, sugar, and protein content. For agitated cultures, the free amino nitrogen (FAN) and ethanol content in the filtrate were also analyzed. Each experiment was conducted in triplicate and presented as mean average values.

After filtering, the retentate was oven-dried at 60 °C, and the dried retentate weight was recorded once it reached constant weight (DW). The protein content of the retentate and filtrate was measured using a bicinchoninic acid assay (BCA) kit (Vazyme, Nanjing, China). The pH before and after cultivation was measured using a pH meter (ST300, OHAUS, Parsippany, NJ, USA). Sucrose, fructose, and glucose were measured using a K-SUFRG enzyme kit, and ethanol was measured using a K-ETOH enzyme kit according to the manufacturer’s instructions (Megazyme, Bray, Ireland). The FAN content of the filtrate was measured using the ninhydrin method [43]. The ninhydrin color reagent consisted of 0.3 g fructose, 0.5 g ninhydrin, 6 g KH_2_PO_4_, and 10 g Na_2_HPO_4_ dissolved in 100 mL of distilled water (pH 6.6). Diluting solution was prepared by dissolving 2 g of potassium iodide in 600 mL of distilled water, which was then added to 400 mL of 96% ethanol. The liquid samples were then diluted 50-fold with distilled water, and then 2 mL of the diluted sample was mixed with 1 mL of ninhydrin color reagent in a test tube. The mixture was then placed in a boiling water bath for 16 min and cooled to room temperature for 20 min. Afterward, 5 mL of the diluting solution was added to each test tube, and the absorbance was measured at 570 nm.

### 2.6. Statistical Analysis

Statistical analysis of the collected data was performed using GraphPad Prism version 9.4.0 (GraphPad Software, Inc., San Diego, CA, USA). One-way ANOVA followed by the Tukey HSD test was carried out to investigate the differences in metabolic activities, biomass, and protein production of *A. oryzae* in different media and conditions. Welch’s *t*-tests were also performed to compare the effect of agitation on retentate concentration and biomass protein content in soy whey or okara cultures. The effect was considered statistically significant if the p-value was less than or equal to the selected significance level (*p*-value < 0.05).

## 3. Results

### 3.1. Enzymatic Activity of A. oryzae

The remarkable enzymatic activities of *A. oryzae* have made it a vital component in the fermentation of soybean-based foods and industrial bioprocessing for many years [34]. These activities, however, can vary greatly between different strains [33]. Therefore, as a first step, we conducted an initial assessment of the strain’s enzymatic activity to gauge its potential for efficient substrate utilization and optimal mycoprotein production. Table 1 shows that *A. oryzae* has amylolytic and proteolytic activity, as evidenced by the clear zones of 9 ± 1.45 mm and 1.3 ± 0.47 mm, respectively. This suggests that the fungus has the ability to utilize starch and protein. However, no lipolytic activity was observed, as indicated by the absence of a precipitation zone, and no cellulase (endo-1,4-β-D-glucanase) activity was detected. A preliminary experiment was also conducted to screen for the *Aspergillus* sp. isolate from soy sauce with the best enzymatic activity. We found that *A. oryzae* (KKC.P0N.A) was more promising due to its high amylase activity (Table S1) and was used for further mycoprotein production.

### 3.2. Metabolic Changes during A. oryzae Cultivation in Okara and Soy Whey Media under Agitated Conditions

**Figure 1:**
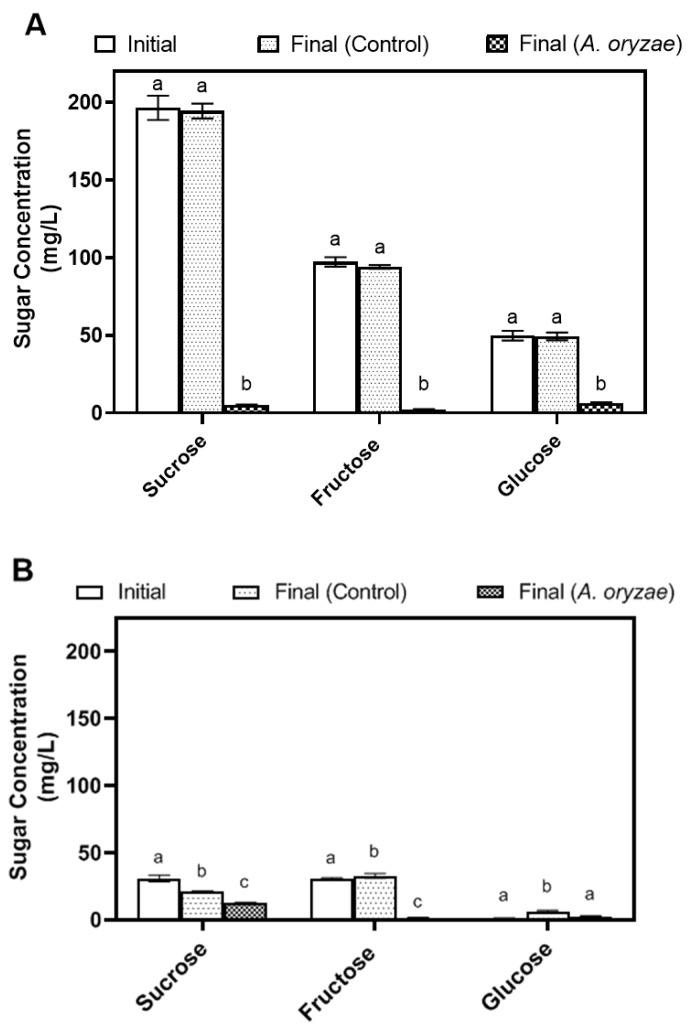
Sugar concentration of (**A**) okara (1:50) and (**B**) soy whey (1:1) media before and after 7 days of incubation at 30 °C with agitation at 100 rpm (inoculum size: 10% *v*/*v*, ~log 6 spores/mL). Different letters indicate significantly different mean values (*p* < 0.05).

During the initial investigation, *A. oryzae* was cultivated with agitation at 100 rpm, a standard practice reported in the literature to enhance aeration and overall mixing [26,44]. To gain deeper insights into its metabolic dynamics during the cultivation, the amounts of sugars (sucrose, fructose, and glucose), ethanol, protein, and free amino nitrogen (FAN) were measured (Figure 1).

Initially, okara contained 196 mg/L sucrose, 97 mg/L fructose, and 49 mg/L glucose (Figure 1A). After cultivation with *A*. *oryzae* (100 rpm), these concentrations significantly decreased to 5.17 mg/L, 2.48 mg/L, and 6.43 mg/L (*p* < 0.0001), respectively, which represents a 96% consumption of the total sugars. In contrast, no significant changes were observed in the control (uninoculated okara). Meanwhile, soy whey started with lower concentrations of sucrose (31 mg/mL), fructose (27.8 mg/L), and glucose (1 mg/L) (Figure 1B). In contrast to okara, we noted a decrease in sucrose to 21.33 mg/L and increases in fructose and glucose to 32.78 mg/L and 6.455 mg/L, respectively, even in the absence of *A. oryzae* (control), suggesting that sucrose underwent spontaneous hydrolysis into its monomers. However, cultivation with *A. oryzae* (100 rpm) led to further decrease in sucrose, fructose, and glucose concentrations, leaving residual amounts of 13.42 mg/L, 1.66 mg/L, and 2.66 mg/L, respectively, which corresponds to 70.7% sugar consumption. Additionally, no ethanol production was detected during cultivation in either medium. The low amount of sugar in the medium might have led to such low ethanol production that it went undetected. Alternatively, the fungi might have consumed all the ethanol by the time measurements were taken [26,27].

**Figure 2:**
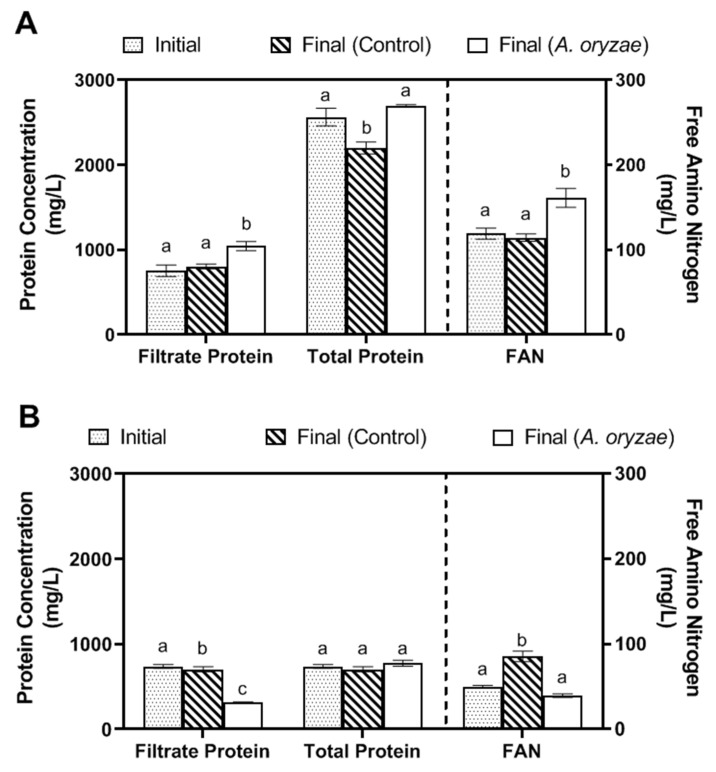

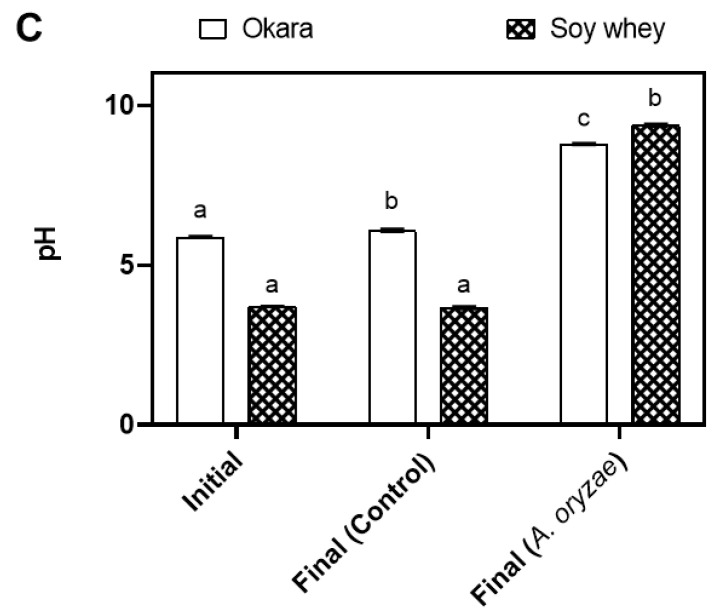
Filtrate, total protein (includes retentate and filtrate protein) concentration, and free amino nitrogen (FAN) (**A**) in okara media and (**B**) soy whey media; (**C**) pH of both okara (1:50) and soy whey (1:1) media before and after 7 days of incubation at 30 °C with agitation at 100 rpm (inoculum size: 10% *v*/*v*, ~log 6 spores/mL). Different letters between the same analytes indicate significantly different mean values (*p* < 0.05).

The ability of *A. oryzae* to metabolize proteins in soy whey and okara media was also evaluated by measuring the total protein and FAN in the filtrate (Figure 2A,B). Regardless of the substrate, the initial and final total protein content remained similar despite changes in protein and FAN concentrations in the filtrate during cultivation. In soy whey cultures, filtrate protein decreased significantly from 787.42 to 317.17 mg/L (*p* < 0.0001), while the control only showed a slight decrease to 734.5 mg/L (Figure 2B). Similarly, FAN levels decreased from 49.93 to 39.52 mg/L (*p* = 0.0013) after 7 days of incubation, in contrast to the control, which exhibited a significant increase in FAN, reaching 88.2 mg/L (*p <* 0.0001). This increase in the control suggests that proteins were breaking down and releasing FAN spontaneously. In soy whey cultures, *A. oryzae* was able to utilize the FAN and filtrate proteins to accumulate the proteins into its biomass, resulting in a final protein content of 777 mg/L, comparable to the initial total protein content of 787.42 mg/L (*p* = 0.858). In okara cultures, the total protein content also increased slightly from 2563 to 2697 mg/L (*p* = 0.1421). In contrast to soy whey cultures, the filtrate protein in okara showed a significant increase from 754.47 to 1044.42 mg/L (*p* = 0.0012), along with a 36% rise in FAN content (~161 mg/L, *p* = 0.0014), whereas the amounts of filtrate protein and FAN in the control remained relatively unchanged (Figure 2A). These results indicate that okara cultures were more focused on breaking down proteins and releasing FAN and extracellular proteins, instead of accumulating the proteins into its biomass.

Furthermore, we observed an unexpected pH increase by the end of incubation in both media. In soy whey, the pH rose significantly from 3.71 to 9.38, while in okara, it increased significantly from 5.88 to 8.79 (both *p* < 0.0001) (Figure 2C). In contrast, the pH in the control samples for both okara and soy whey remained relatively constant.

### 3.3. Biomass Production in Okara and Soy Whey under Agitated Conditions

After 7 days of cultivation in shake flasks (at 100 rpm), the retentate was analyzed to determine the biomass and protein production of *A. oryzae* in both okara and soy whey. For okara, the final retentate included both fungal biomass and residual soy pulp, while the final retentate from soy whey only consisted of fungal biomass. The results revealed a striking contrast in biomass production between the two substrates.

In soy whey, a final fungal biomass of 0.867 g DW/L was obtained in pellet form (Table 2). In contrast, no distinguishable fungal biomass could be observed in okara, making it impossible to determine the biomass yield. Nonetheless, we noted a significant decrease in the dry weight of total retentate in okara by 41.31% (from 13.24 to 7.77 g DW/L, *p* < 0.0001), compared to a mere 3% decrease in the control (to 12.9 g DW/L, *p* = 0.1645), suggesting that *A. oryzae* was capable of breaking down the soy pulp. This significant decrease in retentate, along with changes in sugar, protein, FAN, and pH, suggest that the fungi were metabolically active, though they did not form a distinguishable biomass as observed in soy whey. The increase in filtrate proteins might suggest that fungal biomass potentially passed through the filter membrane during retentate collection (Figure 2A). However, further microscopic observations showed no dispersed mycelia in the filtrate, which leads us to consider the possibility that *A. oryzae* may have formed biomass entangled with the soy pulp, as previously observed by Souza-Filho et al. [45] in starch plant wastewater. This hypothesis was supported by further analysis of the protein content in the okara retentate, which showed an increase in protein content from 0.109 mg/mg DW to 0.213 mg/mg DW after cultivation (*p* < 0.05).

### 3.4. Static Conditions Promote the Production of Biomass in Okara but Reduce It in Soy Whey

The lack of visible fungal biomass formation in okara, despite clear signs of metabolic activity, prompted us to investigate the possible impact of shear stress caused by agitation. It is well documented that agitation at certain speeds can adversely affect biomass yield [46,47]. To gain deeper insights, we also investigated the fungal growth under static conditions.

Agitation clearly affects the biomass production of *A. oryzae* in okara. Without agitation, *A. oryzae* developed a floating biomass on the surface of the culture medium, separate from the soy pulp, with a concentration of 2.02 g DW/L (Table 3). After cultivation, we also noted a 30.8% decrease in soy pulp concentration to 9.16 ± 0.04 g DW/L (*p* < 0.0001), which was less pronounced compared to the decrease in total retentate observed in the agitated cultures. The remaining soy pulp, along with the produced biomass, increased the total retentate concentration in okara to 11.18 g DW/L. This represents a 44% increase compared to agitated culture, which had a total retentate concentration of 7.77 g DW/L (*p* = 0.0054). Additionally, the biomass obtained from static cultivation contained 0.271 mg/mg DW of protein, while protein content in the remaining soy pulp showed no significant change compared to control (0.106 mg/mg DW, *p* = 0.995) (Table 3). This contrasts with the agitated counterparts, in which the protein content in the leftover soy pulp doubled to 0.213 mg/mg DW, likely due to fungal biomass growing and becoming entangled with the soy pulp. In static cultures, the final protein concentration in the okara filtrate increased significantly from 754.47 mg/L to 1554.5 mg/L (*p* < 0.0001), which represents a 48.9% higher protein level compared to okara under agitated conditions (*p =* 0.0002) (Table 4). The significant increase in both retentate and filtrate protein led to a total protein content of 3071 mg/L, which is significantly higher than that of agitated cultures (2696 mg/L, *p* = 0.0019) (Table 4). Interestingly, static cultures showed no significant differences in the amount of sugars consumed (sucrose, fructose, and glucose) and final pH compared to agitated cultures, despite the contrast in biomass and protein production (Table 4).

On the other hand, while static conditions improve biomass production in okara, they resulted in a 9.7% less biomass in soy whey (0.783 g DW/L, *p* = 0.309) compared to the agitated counterparts, despite consuming 11.8% more sugars (51.5 mg/L, *p* = 0.0015) (Table 5). Similarly to okara, soy whey also formed mycelial mats under static conditions. Furthermore, the protein concentration in the filtrate of static soy whey cultures was 30% lower (221.8 ± 3.29 mg/L, *p* = 0.0003) than in agitated cultures, thus resulting in significantly lower total protein content (388 ± 0.64 mg/L, *p* = 0.0002) (Table 5). Additionally, the final pH of soy whey in static culture was lower (pH 8.4) compared to agitated cultures (*p* = 0.0002) (Table 5). Besides lower biomass and filtrate protein production, soy whey under static conditions exhibited significantly lower biomass protein content (0.213 mg/mg DW) compared to the agitated cultures (0.531 mg/mg DW, *p* = 0.0066) (Table 6).

### 3.5. Soy Whey Resulted in a Higher Biomass and Protein Yield per Substrate in Comparison to Okara

The biomass yield per substrate was calculated by dividing the dry weight of the fungal biomass produced by the dry weight of the substrate used. It was found that okara under static conditions produced the lowest biomass yield of 114 mg/g DW substrate (*p* < 0.001) (Table 7). In contrast, soy whey demonstrated approximately three times higher biomass yields, reaching 369 mg/g DW substrate in static cultures and 408 mg/g DW substrate in agitated cultures (*p* = 0.389). When examining biomass protein yield, calculated by dividing the total protein content in the fungal biomass by the dry weight of the substrate, agitated soy whey cultures also led the way with a yield of 216.9 mg/g DW substrate (*p* < 0.0001) (Table 7). This was followed by static soy whey cultures, which showed a protein yield of 78.7 mg/g DW substrate. Okara (static conditions) yielded the lowest biomass protein at 31.07 mg/g DW substrate (*p <* 0.05). These findings highlight the potential of soy whey, especially under agitated conditions, as a promising and cost-effective substrate for mycoprotein production by *A. oryzae*.

## 4. Discussion

The current study is motivated by the research question of whether *A. oryzae* can produce high-protein biomass through cultivation in soybean processing side streams, including okara and soy whey. This question arises from our state-of-the-art knowledge of *A. oryzae*’s performance in producing mycoprotein using various waste products, even outperforming *F. venenatum* in many cases [26,27]. Given *A. oryzae*’s long history of fermenting soybean-based products, utilizing okara and soy whey as substrates could potentially offer a more efficient approach to mycoprotein production and broaden the spectrum of waste products used for mycoprotein while simultaneously reducing the environmental impact.

The key to producing mycoprotein efficiently is the fungi’s enzymatic capacity, including amylase, protease, and lipase. These enzymes break down the carbohydrates, proteins, and lipids in the substrate into simpler molecules, which the fungi can use to produce biomass and protein. The results of the qualitative analysis show that *A. oryzae* has both amylolytic and proteolytic activity (Table 1). The presence of these enzymes would facilitate the mycoprotein production, as fresh okara typically contains ~0.5% starch and 6–33% protein [37,48,49], while soy whey is known to contain 0.1–1.8% proteins and starch (unspecified amount) [38,50,51], depending on factors such as cultivar and processing conditions. Meanwhile, no lipase activity was observed on the Tween 80 agar. However, it is worth noting that this may not necessarily indicate that *A. oryzae* lacks lipase altogether but rather that its specificity towards different substrates can differ [52]. For instance, Griebeler et al. [53] reported that the fungal isolate exhibiting the highest lipolytic activity using soybean bran as a substrate showed the lowest lipolytic clear zones on tributyrin agar. Furthermore, the sensitivity and specificity of the screening method used may vary depending on the type of lipid substrates used (e.g., Tweens, olive oil, and tributyrin) [54,55]. The same reason might explain the lack of observed cellulolytic activity, especially since *A. oryzae* was able to break down okara in both agitated and static conditions. It is also possible that the method of cellulase extraction was not optimal due to the long incubation period and high pH by the end of cultivation, which could have reduced the cellulolytic activity [56,57,58].

Agitation plays a crucial role during submerged fermentation of filamentous fungi by homogenizing the mixture, facilitating heat transfer, and enhancing the concentration of dissolved oxygen in the culture medium [44]. On the other hand, excessive agitation can lead to shear stress which may negatively impact the biomass yield. Our study revealed a distinct contrast in the biomass production trends of *A. oryzae* between the okara and soy whey, with and without agitation. When agitated, *A. oryzae* was capable of forming observable biomass in soy whey, but not in okara media. It was evident that despite the non-observable biomass formation, *A. oryzae* was metabolically active during cultivation in okara, as it consumed higher amounts of sugars compared to soy whey, increased pH of okara, decreased soy pulp mass, and significantly increased the FAN, and protein content in both the filtrate and the retentate. Microscopic observation of the okara filtrate was conducted to determine if the increased protein content was due to the presence of dispersed mycelium in the liquid phase. However, there was no evidence of dispersed mycelium, suggesting that the increased protein content in the okara filtrate might be due to extracellular proteins secreted by *A. oryzae*.

Further cultivation of okara under static conditions suggested that agitation was responsible for the non-observable biomass production. In static okara cultures, *A. oryzae* produced 2.02 g DW/L separable biomass while also showing similar metabolic activities as agitated cultures. Agitation likely affected the fungal morphology by facilitating interactions between fungal cells and soy pulp particles, allowing them to use soy pulp as a support structure and forming a mycelial network throughout the soy pulp. This ultimately results in the biomass becoming intertwined with the soy pulp. A similar phenomenon is commonly observed when cultivating *A. oryzae* in substrates with a high amount of suspended solids, such as starch plant wastewater and bread waste [45,59]. The mycelial network within the soy pulp also creates a higher surface area-to-volume ratio, resulting in greater breakdown of the soy pulp and significantly reducing the amount of leftover soy pulp by the end of agitated cultivation. In contrast, fungal biomass only formed as a mycelial mat on the surface of the media in static cultures and had limited interaction with the soy pulp, resulting in 44% more soy pulp remaining by the end of cultivation. Similar to okara cultures, *A. oryzae* also formed a mycelial mat in soy whey under static conditions, resulting in 0.783 g DW/L biomass. The absence of agitation likely results in limited dissolved oxygen concentration in the culture media [44]. Consequently, the fungi prefer to form a mycelial mat at the air–liquid interface, where oxygen is more readily accessible [60]. This phenomenon has been documented in the submerged cultivation of *Aspergillus niger* HFD5A-1 and FETL-FT3 under static conditions, and, similarly to our soy whey cultures, the mycelial mat turned into spherical pellets as the agitation speed increased [61,62]. Our study showed that agitation led to the formation of spherical pellets in soy whey biomass, resulting in a higher concentration of 0.867 g DW/L compared to static culture. This increased biomass concentration may be attributed to the spherical shape, which offers a greater surface area-to-volume ratio than the mycelial mat morphology observed in static cultures, thereby enhancing nutrient uptake [63]. In addition to increased biomass production, the spherical pellet form of biomass in agitated soy whey also had nearly double the protein content compared to the mycelial mat form in static cultures, reaching 0.531 mg/mg DW (53.1% *w*/*w*). This value surpasses not only the biomass protein produced in okara but also those reported for other substrates, including pea-processing by-product (43–46% *w*/*w*), potato protein liquor (41% *w*/*w*), and spent sulfite liquor (44–48% *w*/*w*) [26,64,65].

Furthermore, the biomass and protein yield per gram of substrate also proved soy whey as an efficient substrate for mycoprotein production compared to okara, regardless of agitation. In fact, the biomass yield of agitated soy whey cultures was found to be double that of the pea-processing by-product and glucose-based semi-synthetic media [26,66]. The higher yield in soy whey compared to okara can be attributed to a combination of factors. Firstly, the formation of pellets in soy whey might have facilitated better nutrient uptake. Secondly, okara’s complex structure may demand more energy from the fungi for substrate breakdown. As a result, the fungi may prioritize producing enzymes to break down the complex okara substrate over producing biomass. This was evident from the significant increase in protein content in the okara filtrate after cultivation, which suggests the presence of extracellular enzymes as previously reported by Pleissner et al. [67]. Lastly, there may be potential inhibitors in okara impacting growth efficiency. These distinct responses highlight the significant impact of substrate composition on fungal growth strategies and underscore the importance of tailoring cultivation conditions to specific media types for optimal biomass production.

However, soy whey still consistently produced lower biomass concentration compared to okara, regardless of agitation. Studies have shown that soy whey typically only contains ~1% carbohydrates (including oligosaccharides), ~1% fats, and ~0.5% proteins and minerals [51,68,69]. Besides the glucose, fructose, and sucrose measured in this study, *A. oryzae* potentially utilized the oligosaccharides, such as stachyose or raffinose to support its growth. Nevertheless, the soy whey media used in this study had roughly 5 and 6 times less reducing sugars and sucrose, respectively, compared to the okara media used in this study. Therefore, the relatively low nutrient content of soy whey may also account for the significantly lower biomass concentrations observed in this study (at least 9- to 180-fold) compared to most studies utilizing other substrates, such as oat flour (6 g DW/L) [31] and vinasse (118 g DW/L) [27]. Furthermore, *A. oryzae* may not have been able to withstand the pH increase (up to ~9.4) during cultivation [44]. Additionally, *A. oryzae* was incubated for 7 days in both substrates which may be excessive. Although Gamarra-Castillo et al. [30] previously observed that *A. oryzae* only entered its stationary phase by day 7–8, other studies have found that biomass production peaks by day 3–4 and decreased with prolonged incubation [70,71]. Therefore, future studies could focus more on optimizing cultivation conditions (ex: reduced incubation time, controlled pH, and optimized substrate concentration) to obtain higher biomass concentration in soy whey [27,70,72].

Furthermore, it is also important to note that tofu by-products, specifically soy whey, are usually discharged with a low pH and relatively high COD, which can have various negative impacts on the environment [38,51]. There is no effective solution currently available to tackle this issue, and as tofu production continues to grow, the amount of waste generated also increases [51,73]. However, this study demonstrates that cultivation by *A. oryzae* can reduce at least 70% of the sugar content and 66–72% of the protein in soy whey, resulting in a reduction in COD. This addresses the environmental concerns associated with soy whey as a waste product. Furthermore, since *A. oryzae* is associated with producing flavorful compounds from soy-based products [74] and other beverages [75,76], the filtrate itself could potentially be utilized as a beverage, which has previously been done by other fungi [77,78]. Despite soy whey exhibiting better performance as a substrate, our study also demonstrated promising results regarding the use of okara for protein production. Cultivating *A. oryzae* in okara still doubled the protein content of the retentate (from 11% *w*/*w* to 21–27% *w*/*w*), regardless of agitation. Furthermore, although okara can be consumed as *gembus* in Indonesia, its protein content is relatively low (~4% *w*/*w*) [79], and *A. oryzae*-fermented okara offers a potentially more nutritious alternative. Additionally, the protein content from both okara cultures is comparable to other studies utilizing different fungi grown in different substrates, such as tempeh wastewater (19.4% *w*/*w*) [44], brewer’s spent grain extract (26.5% *w*/*w*) [80], and apple pomace (21% *w*/*w*) [81]. Alongside mycoprotein production, the filtrate proteins that potentially consist of extracellular enzymes further imply additional applications for okara media by *A. oryzae*. 

Different trends in protein production observed in okara and soy whey highlight *A. oryzae*’s distinct metabolic behavior in response to different substrates, suggesting potential for tailored applications based on specific desired outcomes. Future studies could focus on improving the suitability of soy whey and okara for increased biomass yields and conducting an economic analysis to assess its overall feasibility.

## 5. Conclusions

This study aimed to investigate the potential of *A. oryzae* in generating high-protein biomass through cultivation in soybean processing side streams, specifically okara and soy whey. The study found that the ability of *A. oryzae* to produce biomass and protein from each substrate is influenced not only by the composition of the substrate (e.g., sugars, protein, and free amino nitrogen) but also by the incubation mode (agitated 100 rpm vs. static). Although okara (static) produced the highest biomass concentration (2.02 g DW/L), its efficiency in converting substrate to biomass and protein was relatively low. In contrast, soy whey produced biomass and protein at significantly higher yields, even though its overall biomass production was lower. Additionally, the two substrates exhibited different protein production patterns: okara tended to release protein into the media, while soy whey promoted protein accumulation within the fungal biomass, resulting in up to 53% w/w protein content when agitated. Furthermore, agitation had contrasting effects on the two substrates; it increased biomass and protein production in soy whey but decreased them in okara. In okara, agitation caused fungal biomass to entangle with soy pulp, whereas in soy whey, it led to the formation of spherical pellets. Under static conditions, fungal biomass formed mycelial mats on the media surface in both substrates. These morphological differences likely influenced the contrasting yield between the two substrates in response to agitation. Besides producing high-protein fungal biomass, this approach shows promise in reducing the environmental impact of soy whey by lowering its organic content after cultivation. Future research could focus on improving biomass production by *A. oryzae* in soy whey media by optimizing the cultivation conditions in terms of substrate concentration, pH, incubation time, and aeration. Furthermore, the mycelial biomass could be profiled for its nutritional content (ex: amino acid and fatty acid) and safety in terms of allergenicity, RNA content and mycotoxin production.

## Figures and Tables

**Table 1:** The amylolytic, proteolytic, and lipolytic activities of *A. oryzae* on starch agar, skim milk agar, and Tween 80 agar, respectively, after 24 h of incubation at 30 °C. Cellulase was tested using an enzyme kit at 30 °C; *P. aeruginosa* is the positive control for amylase, protease, and lipase; *Trichoderma* sp. is the positive control for cellulase; n.a. or “not applicable” indicates that the data were not taken.

Microorganism	Zone of Activity (mm)			Activity (U/mL)
	Amylase	Protease	Lipase	Cellulase
*Aspergillus oryzae*	9 ± 1.45	1.3 ± 0.47	0	0
*Pseudomonas aeruginosa*	2 ± 0	6.3 ± 1.25	3 ± 0	n.a.
*Trichoderma* sp.	n.a.	n.a.	n.a.	3.64 ± 0.12

**Table 2:** Characteristics of retentate produced from okara (1:50) and soy whey (1:1) media after 7 days incubation at 30 °C with agitation at 100 rpm. Different letters between different rows indicate significantly different mean values (*p* < 0.05). Results presented with “n.d.” mean the data could not be determined, while “n.a.” means that the data was not applicable.

	Retentate Concentration (g DW/L)			Retentate Protein Content
Media	Fungal Biomass	Residual Soy Pulp	Total Retentate	(mg/mg DW)
Okara (control)	n.d.	12.86 ± 0.21	12.86 ± 0.21 ^a^	0.109 ± 0.01 ^a^
Okara (*A. oryzae*)	n.d.	n.d.	7.77 ± 0.4 ^b^	0.213 ± 0.04 ^b^
Soy Whey (control)	n.a.	n.a.	n.a.	n.a.
Soy Whey (*A. oryzae*)	0.867 ± 0.09	n.a.	0.867 ± 0.09 ^c^	0.531 ± 0.04 ^c^

**Table 3:** Characteristics of the retentate (including biomass and soy pulp) produced from okara (1:50) media after 7 days of incubation at 30 °C, either with agitation at 100 rpm or under static conditions. Different letters between different columns indicate significantly different mean values obtained from Welch’s *t*-test (*p* < 0.05). Results presented with “n.d.” mean the data could not be determined.

Parameters	Agitated	Static
Biomass concentration (g DW/L)	n.d.	2.02 ± 0.06
Residual soy pulp concentration (g DW/L)	n.d.	9.16 ± 0.04
Total retentate concentration (g DW/L)	7.77 ± 0.4 ^a^	11.18 ± 0.08 ^b^
Biomass protein (mg/mg DW)	n.d.	0.271 ± 0.008
Soy pulp protein (mg/mg DW)	n.d.	0.106 ± 0.01
Total retentate protein (mg/L)	1653 ± 32.6 ^a^	1518 ± 16.5 ^b^

**Table 4:** Characteristics of okara media (1:50) before and after 7 days of incubation at 30 °C, either with agitation at 100 rpm or under static conditions. Different letters between different columns indicate significantly different mean values (*p* < 0.05).

Parameters	Initial	Final Agitated	Final Static
pH	5.88 ± 0.03 ^a^	8.79 ± 0.02 ^b^	8.78 ± 0.04 ^b^
Glucose (mg/L)	49.89 ± 2.58 ^a^	6.43 ± 0.5 ^b^	2.08 ± 0.11 ^b^
Fructose (mg/L)	97.24 ± 2.5 ^a^	2.48 ± 0.15 ^b^	3.4 ± 0.33 ^b^
Sucrose (mg/L)	196.41 ± 6.35 ^a^	5.17 ± 0.33 ^b^	4.13 ± 0.29 ^b^
Filtrate Protein (mg/L)	754 ± 54.3 ^a^	1044 ± 43.7 ^b^	1555 ± 107 ^c^
Total Protein Content (mg/L) *	2529 ± 82.6 ^a^	2696 ± 11 ^a^	3071 ± 61.4 ^b^

* Total Protein Content refers to the combination of filtrate and retentate protein.

**Table 5:** Characteristics of soy whey (1:1) media before and after 7 days incubation at 30 °C, either with agitation at 100 rpm or in static conditions. Different letters between different columns indicate significantly different mean values (*p* < 0.05).

Parameters	Initial	Final Agitated	Final Static
pH	3.71 ± 0.01 ^a^	9.38 ± 0.03 ^b^	8.4 ± 0.17 ^c^
Glucose (mg/L)	1.35 ± 0.11 ^a^	2.63 ± 0.4 ^b^	1.89 ± 0.42 ^ab^
Fructose (mg/L)	27.82 ± 1.95 ^a^	1.67 ± 0.16 ^b^	1.18 ± 0.03 ^b^
Sucrose (mg/L)	31.29 ± 2.6 ^a^	13.42 ± 0.12 ^b^	8.94 ± 0.66 ^b^
Filtrate Protein (mg/L)	787 ± 16.9 ^a^	317 ± 4.25 ^b^	222 ± 3.29 ^c^
Total Protein Content (mg/L) *	787 ± 16.9 ^a^	777 ± 29 ^b^	388 ± 0.64 ^b^

* Total Protein Content refers to the combination of filtrate and retentate protein.

**Table 6:** Characteristics of retentate (fungal biomass only) produced from soy whey (1:1) media after 7 days incubation at 30 °C, either with agitation at 100 rpm or in static conditions. Different letters between different columns indicate significantly different mean values (*p* < 0.05).

Parameters	Agitated	Static
Biomass concentration (g DW/L)	0.867 ± 0.09 ^a^	0.783 ± 0.02 ^a^
Biomass protein (mg/mg DW)	0.531 ± 0.004 ^a^	0.213 ± 0.005 ^b^
Biomass protein (mg/L)	459.8 ± 33 ^a^	166.9 ± 3.6 ^b^

**Table 7:** Biomass and protein yield per substrate obtained from okara (1:50) and soy whey (1:1) media after 7 days of incubation at 30 °C either with agitation at 100 rpm or in static conditions. Different letters between different columns indicate significantly different mean values (*p* < 0.05). Results presented with “n.d.” mean the data could not be determined.

Parameters	Okara		Soy Whey	
	Agitated	Static	Agitated	Static
Biomass yield/substrate (mg DW/g DW)	n.d.	114.7 ± 3.68 ^a^	408.8 ± 47.1 ^b^	369.2 ± 9.9 ^b^
Biomass protein yield/substrate (mg DW/g DW)	n.d.	31.07 ± 0.88 ^a^	216.9 ± 15.7 ^b^	78.73 ± 1.68 ^c^

## Data Availability

The original contributions presented in the study are included in the article and Supplementary Material, further inquiries can be directed to the corresponding author.

## Supplementary Materials

The following supporting information can be downloaded at: https://www.mdpi.com/article/10.3390/jof10080555/s1, Table S1. The amylolytic, proteolytic, and lipolytic activities of *Aspergillus* isolates on starch agar, skim milk agar, and Tween 80 agar, respectively, after 24 hours of incubation at 30℃. Cellulase was tested using an enzyme kit at 30 °C; *P. aeruginosa* is the positive control for amylase, protease, and lipase; *Trichoderma* sp. is the positive control for cellulase; n.a. or “not applicable” indicates that the data was not taken.
