# Supplementary material for: Exploring the Potential of Aspergillus oryzae for Sustainable Mycoprotein Production Using Okara and Soy Whey as Cost-Effective Substrates
Source: J Fungi (Basel). 2024 Aug 7;10(8):555. doi: 10.3390/jof10080555 (PMC11355589; doi:10.3390/jof10080555)
Supplement: Supplementary file 1 [file jof-10-00555-s001.zip › jof-2992226-supplementary.pdf]

Supplementary Materials

Table S1. The amylolytic, proteolytic, and lipolytic activities of *Aspergillus* isolates on starch agar, skim milk agar, and Tween 80 agar, respectively, after 24 hours of incubation at 30℃. Cellulase was tested using an enzyme kit at 30℃; *P. aeruginosa* is the positive control for amylase, protease, and lipase; *Trichoderma* sp. is the positive control for cellulase; n.a. or "not applicable" indicates that the data was not taken.

**Microorganism**	**Zone of Activity (mm)**			**Activity (U/mL)**
	**Amylase**	**Protease**	**Lipase**	**Cellulase**
KKC.P0N.A	9±1.45	1.33±0.47	0	0
KM1.P5NC	6.66±1.44	1.37±0.52	0	0
KK.P1ONJ	4.76±1.31	0.83±0.24	0	0
Pseudomonas aeruginosa	2±0	6.3±1.25	3±0	n.a.
Trichoderma sp.	n.a.	n.a.	n.a.	3.64±0.12
